# Supplementary material for: Refining Evaluation of Bone Mass and Adipose Distribution in Dunnigan Syndrome
Source: Int J Mol Sci. 2023 Aug 23;24(17):13118. doi: 10.3390/ijms241713118 (PMC10488191; doi:10.3390/ijms241713118)
Supplement: Supplementary file 1 [file ijms-24-13118-s001.zip › ijms-2535286-supplementary.pdf]

SUPPLEMENTARY DATA

Table S1: Linear regression results between laboratorial, densitometric and magnetic resonance variables before and after adjustment for sex, age, BMI and diabetes mellitus

	Unadjusted			Adjusted		
	**β effect**	**CI**	**p-value**	**β effect**	**CI**	**p-value**
FG x LS BMD	4.24	-10.21:18.68	0.56	8.99	-0.85:18.83	0.07
FG x TH BMD	2.99	-12.93:18.91	0.71	9.57	-2.60:21.75	0.12
FG x FN BMD	3.96	-12.08:19.99	0.62	12.18	0.71:23.65	<0.05
FG x 1/3 radius BMD	-25.96	-45.06:-6.85	<0.01	-4.34	-28.21:19.52	0.71
HbA1c x 1/3 radius BMD	-1.20	-0.76:-1.25	<0.01	-0.37	-0.39:0.24	0.64
HOMA-IR x 1/3 radius BMD	-0.43	-0.04:89.5	0.52	-0.90	-0.03:0.57	0.05
OCN x LS BMD	-1.01	-2.06:0.04	0.06	-1.04	-1.99:-0.09	<0.05
OCN x TH BMD	-0.67	-1.85:0.52	0.26	-0.84	-2.03:0.36	0.17
OCN x FN BMD	-0.81	-2.00:037	0.17	-0.73	-1.89:0.44	0.21
FG x TBS	-26.26	-48.47:-4.06	<0.05	5.95	-15.41:27.32	0.58
HbA1c x TBS	-2.26	-0.92:3.30	<0.01	-0.09	-0.21:0.75	0.64
OCN x TBS	0.13	-1.67:1.93	0.89	-0.30	-2.39:1.80	0.78
HOMA-IR x TBS	-2.95	-0.04:818.47	0.25	-7.92	-0.15:-320.18	<0.01
EMCL x TBS	-3577.45	-598.14:-13308.65	<0.01	-1410.09	-135.31:-4842.39	<0.05
EMCL x LS BMD	-681.17	-299.67:-706.46	<0.05	-250.57	-68.33:-392.41	<0.05
EMCL x TH BMD	-774.27	-328.41:-925.64	<0.01	-300.16	-76.91:-576.98	<0.05
EMCL x FN BMD	-681.09	-297.10:-667.18	<0.05	-236.45	-59.36:26.01	0.05
EMCL x 1/3 radius BMD	-983.44	-298.86:-1003.47	<0.05	-520.98	-72.23:4516.03	0.13
LS BMAT x TBS	-4.74	-9.54:0.06	0.05	-3.10	-9.15:2.95	0.30
IMCL x TBS	-271.22	-25.07:6576.19	0.22	-66.65	-3.04:13203.62	0.42
IMCL/EMCL x TBS	1.62	-3.93:7.17	0.56	2.35	-5.37:10.06	0.54
IMCL x LS BMD	2.55	-11.4:864.38	0.96	2.72	2.28:901.57	0.90
IMCL x TH BMD	18.13	-8.9:1202.92	0.70	19.11	-1.6:1916.04	0.47
LS BMAT x LS BMD	-1.64	4.93:1.65	0.32	-1.49	-4.62:1.63	0.33
LS BMAT x TH BMD	-0.79	-4.60:3.02	0.68	-0.75	-4.78:3.28	0.71

Abbreviations: FG, fasting glucose; BMD, bone mineral density; LS, lumbar spine; TH, total hip; FN, femoral neck; HbA1c, glycated hemoglobin; HOMA-IR, Homeostasis Model Assessment; OCN, osteocalcin; EMCL, extra-myocyte lipids; IMCL, intra-myocyte lipids; LS BMAT, lumbar spine bone marrow adipose tissue
